# Investigation and Restoration of BEST1 Activity in Patient-derived RPEs with Dominant Mutations

**DOI:** 10.1038/s41598-019-54892-7

**Published:** 2019-12-13

**Authors:** Changyi Ji, Yao Li, Alec Kittredge, Austin Hopiavuori, Nancy Ward, Peng Yao, Yohta Fukuda, Yu Zhang, Stephen H. Tsang, Tingting Yang

**Affiliations:** 1 Department of Pharmacology and Physiology, University of Rochester, School of Medicine and Dentistry, Rochester, NY 14642 USA; 2 Department of Ophthalmology, Columbia University, New York, NY 10032 USA; 3 Aab Cardiovascular Research Institute, Department of Medicine, University of Rochester, School of Medicine & Dentistry, Rochester, NY 14586 USA; 4 Division of Advance Pharmaco-Science, Graduate School of Pharmaceutical Science, Osaka University, Yamadaoka 1-6, Suita Osaka, 565-0871 Japan; 5 Jonas Children’s Vision Care, and Bernard & Shirlee Brown Glaucoma Laboratory, Departments of Ophthalmology and Pathology & Cell Biology, Edward S. Harkness Eye Institute, Columbia Stem Cell Initiative, New York Presbyterian Hospital/Columbia University, New York, NY 10032 USA

**Keywords:** Chloride channels, Patch clamp, Gene therapy, Hereditary eye disease, Retinal diseases

## Abstract

BEST1 is a Ca^2+^-activated Cl^−^ channel predominantly expressed in retinal pigment epithelium (RPE), and over 250 genetic mutations in the *BEST1* gene have been identified to cause retinal degenerative disorders generally known as bestrophinopathies. As most *BEST1* mutations are autosomal dominant, it is of great biomedical interest to determine their disease-causing mechanisms and the therapeutic potential of gene therapy. Here, we characterized six Best vitelliform macular dystrophy (BVMD)-associated *BEST1* dominant mutations by documenting the patients’ phenotypes, examining the subcellular localization of endogenous BEST1 and surface Ca^2+^-dependent Cl^−^ currents in patient-derived RPEs, and analyzing the functional influences of these mutations on BEST1 in HEK293 cells. We found that all six mutations are loss-of-function with different levels and types of deficiencies, and further demonstrated the restoration of Ca^2+^-dependent Cl^−^ currents in patient-derived RPE cells by WT *BEST1* gene supplementation. Importantly, *BEST1* dominant and recessive mutations are both rescuable at a similar efficacy by gene augmentation via adeno-associated virus (AAV), providing a proof-of-concept for curing the vast majority of bestrophinopathies.

## Introduction

Genetic mutation of the human *BEST1* gene causes bestrophinopathies, which consist of a spectrum of retinal degeneration disorders including Best vitelliform macular dystrophy (BVMD)^1,2^, autosomal recessive bestrophinopathy (ARB)^3^, adult-onset vitelliform dystrophy (AVMD)^4,5^, autosomal dominant vitreoretinochoroidopathy (ADVIRC)^6^, and retinitis pigmentosa (RP)^7^. BVMD, featuring an early-onset and debilitating form of central macular degeneration, is the most common bestrophinopathy. Due to abnormalities in the fluid and/or electrolyte homeostasis between the RPE and photoreceptor outer segments^8^, the disease leads to the formation of serous retinal detachment and lesions that resemble egg yolk, or vitelliform, while rod and cone photoreceptor function remains unaffected. All types of bestrophinopathies, except for ARB, result from autosomal dominant mutation of *BEST1*. Patients are susceptible to untreatable, progressive vision loss, which significantly deteriorates life quality. Therefore, understanding the mechanisms of *BEST1* disease-causing mutations and designing strategies to restore the damaged cellular function are critical for developing treatments for bestrophinopathies.

The protein encoded by the *BEST1* gene is a Cl^−^ channel named BESTROPHIN1 (BEST1), which is activated in response to intracellular Ca^2+^ and conducts Ca^2+^-dependent Cl^−^ current on the cell membrane of retinal pigment epithelium (RPE)^1,2,9,10^. Consistently, Ca^2+^-dependent Cl^−^ current has been suggested to generate a critical visual response upon light exposure, namely light peak (LP)^11–13^, which is defective in almost all *BEST1*-mutated patients as shown by electrooculography (EOG)^14,15^. This BEST1- Cl^−^ current- LP correlation suggests gene supplementation as a promising approach for curing bestrophinopathies. Indeed, we reported that the impaired Cl^−^ current in RPE derived from an ARB patient bearing a *BEST1* recessive mutation was rescuable by baculovirus (BV) -mediated supplementation of the WT *BEST1* gene^9^. Moreover, a recent study in canine models demonstrated that the retinal abnormalities caused by recessive mutation of *BEST1* can be corrected by adeno-associated virus (AAV) -mediated subretinal *BEST1* gene augmentation^16^. However, the rescue efficacy of gene augmentation for *BEST1* dominant mutations is still unknown. This is a very important question because firstly, most of *BEST1* mutations are dominant, and secondly, it will determine whether disruption/suppression of the dominant mutant allele is necessary in therapeutic interventions. In principle, the excess of WT BEST1 could overwhelm the mutant BEST1 despite the latter being dominant over the former at a 1:1 ratio. As canines do not have *BEST1* dominant mutation genotypes while *Best1* knockout mice do not show any retinal phenotype or Cl^−^ current abnormality^17,18^, patient-derived RPEs offer a more relevant model for testing the rescue of *BEST1* dominant mutations.

Here, we analyzed six *BEST1* dominant mutations from BVMD patients, namely p.A10T, p.R218H, p.L234P, p.A243T, p.Q293K and p.D302A, using clinical examinations, patient-derived RPEs, electrophysiological recordings and structural models. Our results showed that these mutations are all loss-of-function with complete or partial deficiency of channel activity, while some of them affect the subcellular localization and/or Ca^2+^-sensitivity of BEST1. Remarkably, defective Ca^2+^-dependent Cl^−^ currents in patient-derived RPE cells were restored by virus-mediated supplementation of the WT *BEST1* gene in a time- and dose-dependent manner. Moreover, both dominant and recessive mutations of *BEST1* are rescuable at a similar efficacy, and both BV and AAV can be used as the vector for gene delivery. Together, our findings underscore the great potential of gene augmentation therapy in treating bestrophinopathies, including those caused by *BEST1* dominant mutations.

## Results

### Retinal phenotypes of six BVMD patients with different *BEST1* mutations

**Figure 1:**
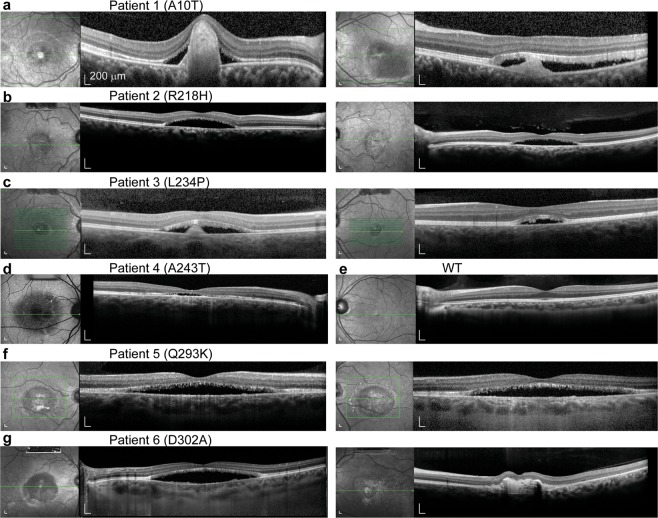
Clinical phenotypes of six patients with *BEST1* mutations. (**a**–**c**) Fundus infrared reflectance image and Spectral Domain Optical Coherence Tomography (SDOCT) of the maculae from patient 1 (**a**), patient 2 (**b**) and patient 3 (**c**), right and left eyes, respectively. (**d**) Fundus infrared image and SDOCT of patient 4 right eye. (**e**) Fundus infrared reflectance image and SDOCT of a WT left eye. (**f**,**g**) Fundus infrared reflectance image and SDOCT of the maculae from patient 5 (**f**) and patient 6 (**g**), right and left eyes, respectively.

We examined six BVMD patients from unrelated families. Generalized retinal dysfunction was found in all six patients. Fundus autofluorescence imaging and optical coherence tomography (OCT) revealed vitelliform lesions located in the subretinal space, as well as serous retinal detachments and cystic fluid in the maculae area (Fig. 1 and Supplementary Fig. S1). Unlike *BEST1* recessive patients, whose electroretinography (ERG) and EOG results are significantly different from WT people^9^, BVMD patients display normal ERG but abnormal EOG results (Supplementary Fig. S2)^19^.

Patient 1, a 6-year-old otherwise healthy girl with a heterozygous c.28 G > A; p.A10T mutation, showed reduced visual acuities at 20/80 and 20/125 in the right and left eye, respectively (Table 1). Large-area, massive vitelliform lesion was observed in the maculae of both eyes, and presented hypo-autofluorescence on fundus autofluorescence imaging. The right macula had subretinal fibrosis. OCT revealed retinal detachments in both eyes with raised fibrotic mounds in the center of the vitelliform lesion and abnormal, elongated photoreceptor outer segments. Intraretinal fluid was also observed from OCT in both eyes (Fig. 1a and Supplementary Fig. S1). Patient 2, a 52-year-old otherwise healthy man with a heterozygous c.653 G > A; p.R218H mutation, showed reduced visual acuities at 20/100 and 20/50 in the right and left eye, respectively (Table 1). OCT detected a thin photoreceptor layer in each eye, extensive subretinal serous fluid and probable vitelliform lesion (Fig. 1b and Supplementary Fig. S1). Patient 3, a 7-year-old otherwise healthy boy with a heterozygous c.701 T > C; p.L234P mutation, showed reduced visual acuities at 20/25 in both eyes (Table 1). Color fundus picture and OCT showed standard boundaries-cleared yellowish vitelliform lesion in the macular area of both eyes, as well as subretinal serous fluid and retinal outer segment debris (Fig. 1c and Supplementary Fig. S1). Patient 4, a 61-year-old otherwise healthy woman with a heterozygous c.728 G > A; p.A243T mutation, showed reduced visual acuity at 20/100 in the right eye (Table 1). No data were recorded for her left eye, which has no light perception due to previous intraocular trauma with a foreign body. The vitelliform material in her right eye displayed hyper-autofluorescence in fundus autofluorescence imaging and was detected in the macular area by OCT (Fig. 1d and Supplementary Fig. S1). Her EOG testing consisted of noisy background, and there was a decrease of light rise in both eyes (Supplementary Fig. S2). Patient 5, a 44-year-old otherwise healthy man with a heterozygous c.877 C > A; p.Q293K mutation, showed reduced visual acuities at 20/50 in both eyes (Table 1). Hyper-autofluorescence of yellowish subretinal vitelliform deposits were observed in both maculae areas (Fig. 1f and Supplementary Fig. S1). EOG results showed loss of light rise (Supplementary Fig. S2). Patient 6 is a 19-year-old otherwise healthy man with a heterozygous c.905 A > C; p.D302A mutation, whose best corrected vision is unknown (Table 1). Vitelliform lesion with autofluoresence, serous retinal detachments and cystic fluid were found in both maculae areas (Fig. 1g and Supplementary Fig. S1).

**Table 1:** Patient information.

Patient #	Age	Gender	Mutation	Vision (OD)	Vision (OS)
1	6	F	A10T	20/80	20/125
2	52	M	R218H	20/100	20/50
3	7	M	L234P	20/25	20/25
4	61	F	A243T	20/100	NA
5	44	M	Q293K	20/50	20/50
6	19	M	D302A	NA	NA

All patients have mutations in the *BEST1* gene.

Age: patients’ age at their visit; Vision: best corrected vision; OD: right eye; OS: left eye.

### *BEST1* dominant mutations impair the channel activity of BEST1

**Figure 2:**
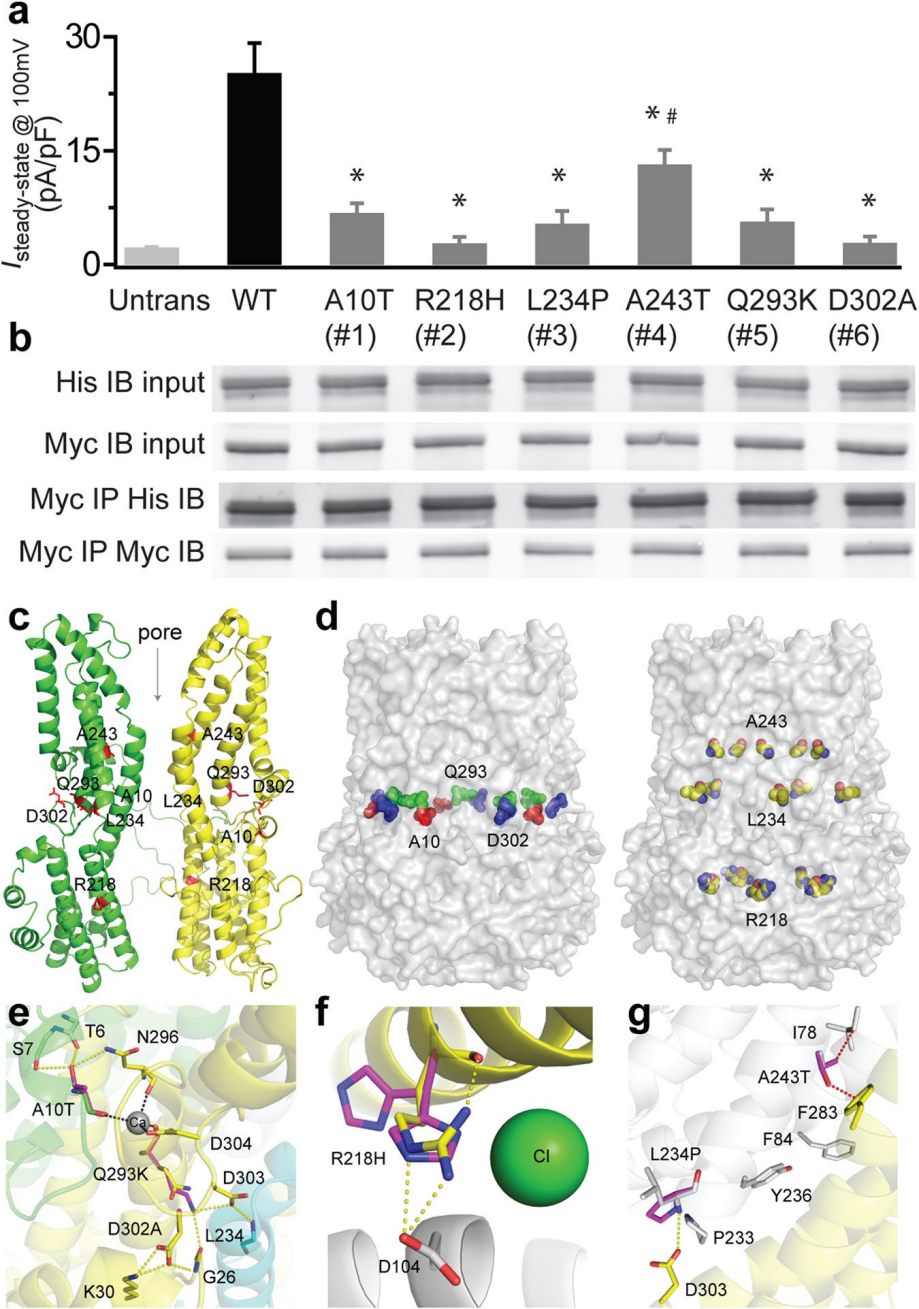
Disease-causing mechanisms of BEST1 mutations. (**a**) Bar chart showing population steady-state current densities at 100 mV for transiently expressed BEST1 WT and mutants in HEK293 cells at 1.2 μM [Ca^2+^]_i_; n = 5–6 for each point. *P = 4 × 10^-5^ (A10T), 7 × 10^−4^ (R218H), 2 × 10^−3^ (L234P), 3 × 10^−2^ (A243T), 2 × 10^−4^ (Q293K), 2 × 10^−3^ (D302A), compared to WT, respectively, and ^#^*P* = 6 × 10^−4^ for A243T compared to untransfected cells, using two-tailed unpaired Student *t* test. (**b**) WT or mutant BEST1-YFP-His was co-transfected with WT BEST1-CFP-Myc to HEK293 cells, and detected by immunoblotting directly in cell lysate (input) or after co-immunoprecipitation. The full-length blots are shown in Fig. S6. All error bars in this figure represent s.e.m. (**c**) Ribbon diagram of two oppositely facing (144°) protomers of a BEST1 pentamer are shown with the extracellular side on the top. The side chains of critical residues are in red. (**d**) Location of the patient mutations in relationship to the channel pore, as viewed from the side. A10 (red), Q293 (green) and D302 (blue) are colored differently. R218, L234 and A243 are colored by atoms. (**e**) Possible interactions of the mutated residues. Each monomeric unit is drawn by a different color and the mutated residues are colored in magenta. Coordination bonds and possible hydrogen bonds are illustrated by dotted black and yellow lines, respectively. (**f**) The effect of replacing R218 with H. Possible conformations of H218 without steric hindrance are shown by magenta sticks. Each monomeric unit is drawn by a different color. Hydrogen bonds are illustrated by dotted yellow lines. (**g**) The effect of replacing A243 and L234. Each monomeric unit is drawn by a different color and the mutated residues are colored in magenta. Possible van der Waals contact or steric hindrance are indicated by dotted red lines. A possible hydrogen bond is illustrated by a dotted yellow line.

To test the influence of the mutations on BEST1 channel activity, WT and six mutant BEST1 channels were individually introduced into HEK293 cells, which do not have any endogenous Ca^2+^-activated Cl^−^ channel on the plasma membrane (Fig. 2a)^9^. HEK293 cells expressing BEST1 mutants displayed significantly smaller currents than WT at 1.2 μM [Ca^2+^]_i_ (Fig. 2a and Supplementary Fig. S3), where cells expressing WT BEST1 conducted peak current amplitude^20^. In particular, five mutants (A10T, R218H, L234P, Q293K and D302A) yielded tiny currents with no significant difference from untransfected cells (Fig. 2a and Supplementary Fig. S3), while the A243T mutant conducted robust currents significantly larger than those from untransfected cells but significantly smaller than those from WT BEST1 (Fig. 2a and Supplementary Fig. S3). Therefore, these six dominant mutations lead to a complete or partial loss of the BEST1 channel activity.

### *BEST1* dominant mutants interact with WT

The BEST1 channel is a pentamer. To test if the interaction between BEST1 monomers is affected by any of the dominant mutations, we overexpressed mutant BEST1-YFP-His and WT BEST1-CFP-Myc in HEK293 cells, followed by immunoprecipitation with an antibody against Myc and immunoblotting with antibodies against His and Myc, respectively. All six dominant mutants were expressed at similar levels to that of WT BEST1 after transient transfection, and retained the interaction with WT BEST1 (Fig. 2b).

### Structural influence of *BEST1* mutations

To seek the structural bases of the functional results, we analyzed a BEST1 homology model generated from the structure of chicken bestrophin1 (cBEST1) (Fig. 2c,d)^9,21–23^, which has 74% sequence identity with BEST1. In this model, A10, Q293 and D302 are located in the Ca^2+^-binding sites on the N-terminus or between S4a and S4b (Fig. 2c,d). The A10T and Q293K mutations are predicted to impair the binding of Ca^2+^, which is coordinated by the acidic side chains of the Ca^2+^-clasp and the backbone carbonyl oxygens from A10 and Q293 (Fig. 2e)^21^: the A10T mutation might make additional hydrogen bonds with surrounding residues including N296, one of the Ca^2+^ ligands; the replacement of Q293 with a lysine residue would form new interactions including a hydrogen bond with G26 and an electrostatic interaction with D303 on the Ca^2+^ binding loop. As D303 forms a hydrogen bond with L234 on the transmembrane helix S3b of the adjacent molecule, which contains residues controlling channel gating, the Q293K mutation also seems to have an indirect influence on channel gating. The D302A mutation changes a negative residue to a hydrophobic residue in the carboxylate loop, potentially weakening the binding of Ca^2+^ to the channel (Fig. 2e). Moreover, this mutation may destabilize the Ca^2+^ binding loop since it presumably eliminates a hydrogen bond with G26 and an electrostatic interaction with K30. Therefore, the A10T, Q293K and D302A mutations may prohibit channel activation by diminishing Ca^2+^ binding, which is absolutely required for BEST1 to conduct current^9,24^.

R218 is localized on the alpha helix S3a (Fig. 2c,d), which falls on a putative Cl^−^ binding site in the channel inner cavity (Fig. 2f)^21^. So, the R218H mutation may decrease the local concentration of anions at the permeation pore, thereby disrupting channel activity. The model structure of the R218H mutant also predicts more flexibility of H218 compared to R218 because of histidine’s smaller side chain. Furthermore, H218 lacks a hydrogen bond with its own carbonyl O atom and is presumably located farther from D104 on the adjacent molecule compared to R218. Hence, the R218H mutant might destabilize the local structure and weaken the interaction between monomers.

L234 and A243 are localized on the transmembrane alpha helix S3b (Fig. 2c,d), which contains multiple residues (e.g. P233 and Y236) critical for channel gating (Fig. 2g)^25,26^. In fact, the model structures predict that the L234P mutation cannot form a hydrogen bond with D303 from the adjacent molecule, while the A243T mutation may have steric hindrances with I78 in the same molecule and F283 from the adjacent molecule (Fig. 2g). Moreover, the L234P mutant may have a highly flexible structure around the mutation site due to consecutive proline residues.

### Mutations in residues involved in Ca^2+^ binding disrupt membrane localization of BEST1

**Figure 3:**
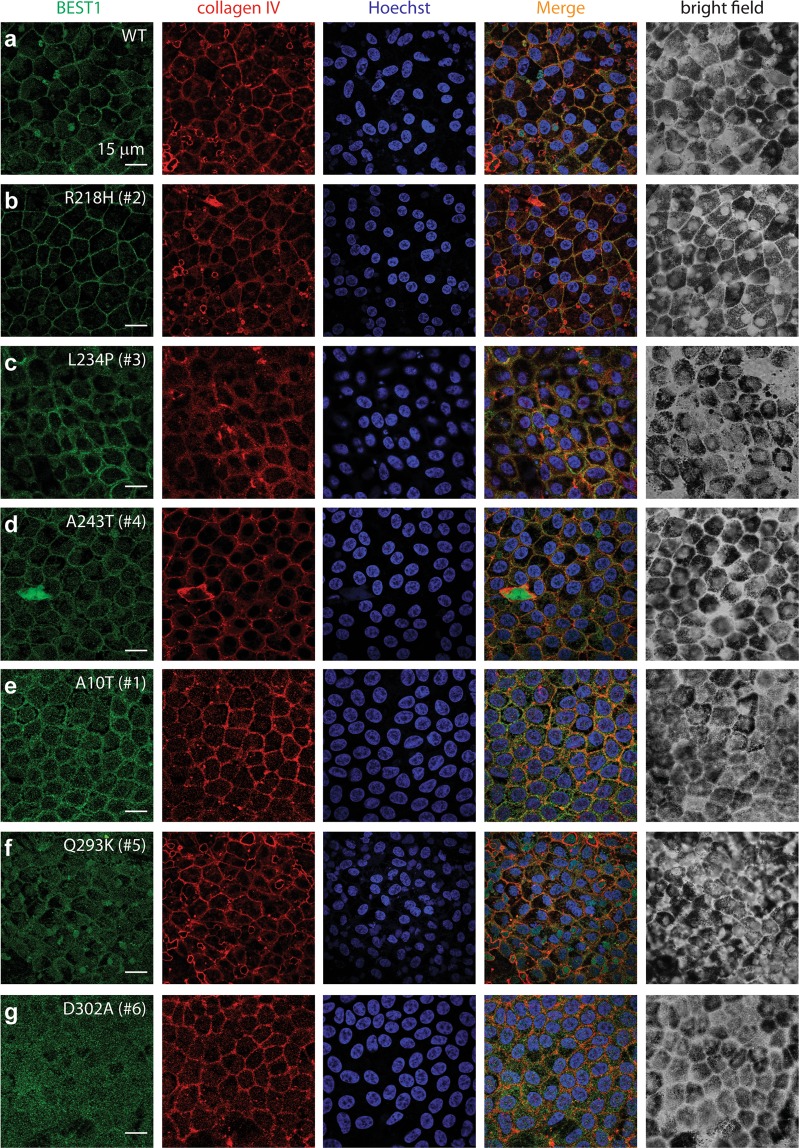
Subcellular localization of WT and mutant BEST1 in iPSC-RPEs. Confocal images showing the co-staining of BEST1, Collagen IV and Hoechst in iPSC-RPEs derived from a WT donor or patients.

To directly examine the physiological impact of the six patient-specific *BEST1* dominant mutations, induced pluripotent stem cells (iPSCs) were reprogrammed from the patients’ skin cells and then differentiated to RPE cells (iPSC-RPEs)^27^. The RPE status of the cells was confirmed by morphological signatures including intracellular pigment and hexagonal shape (Fig. 3). RPE-specific marker proteins RPE65 (retinal pigment epithelium-specific 65 kDa protein) and CRALBP (cellular retinaldehyde-binding protein) were well expressed in iPSC-RPEs derived from a *BEST1* WT donor and the patients as shown by immunoblotting (Supplementary Fig. S4), confirming the mature status of all iPSC-RPEs. Moreover, all six patient-derived iPSC-RPEs showed a similar overall BEST1 expression level compared to that in iPSC-RPE derived from the *BEST1* WT donor (Supplementary Fig. S4), indicating that none of the six mutations impairs the global protein expression of the channel.

We then examined the subcellular localization of BEST1 in iPSC-RPEs by immunostaining. R218H, L234P and A243T displayed normal BEST1 signals on the plasma membrane just like the WT (Fig. 3a–d). By contrast, all three mutations of residues involved in Ca^2+^ binding exhibited deficiency in the membrane targeting of BEST1: D302A has the strongest phenotype with a complete loss of BEST1 antibody staining signal on the plasma membrane, while A10T and Q293K both partially lost membrane localization of BEST1 (Fig. 3e–g). Consistently, immunoblotting showed decreased levels of the A10T, Q293K and D302A mutant proteins on the cell membrane (Supplementary Fig. S4).

### Deficient Ca^2+^-dependent Cl^−^ current in iPSC-RPEs bearing *BEST1* dominant mutations

**Figure 4:**
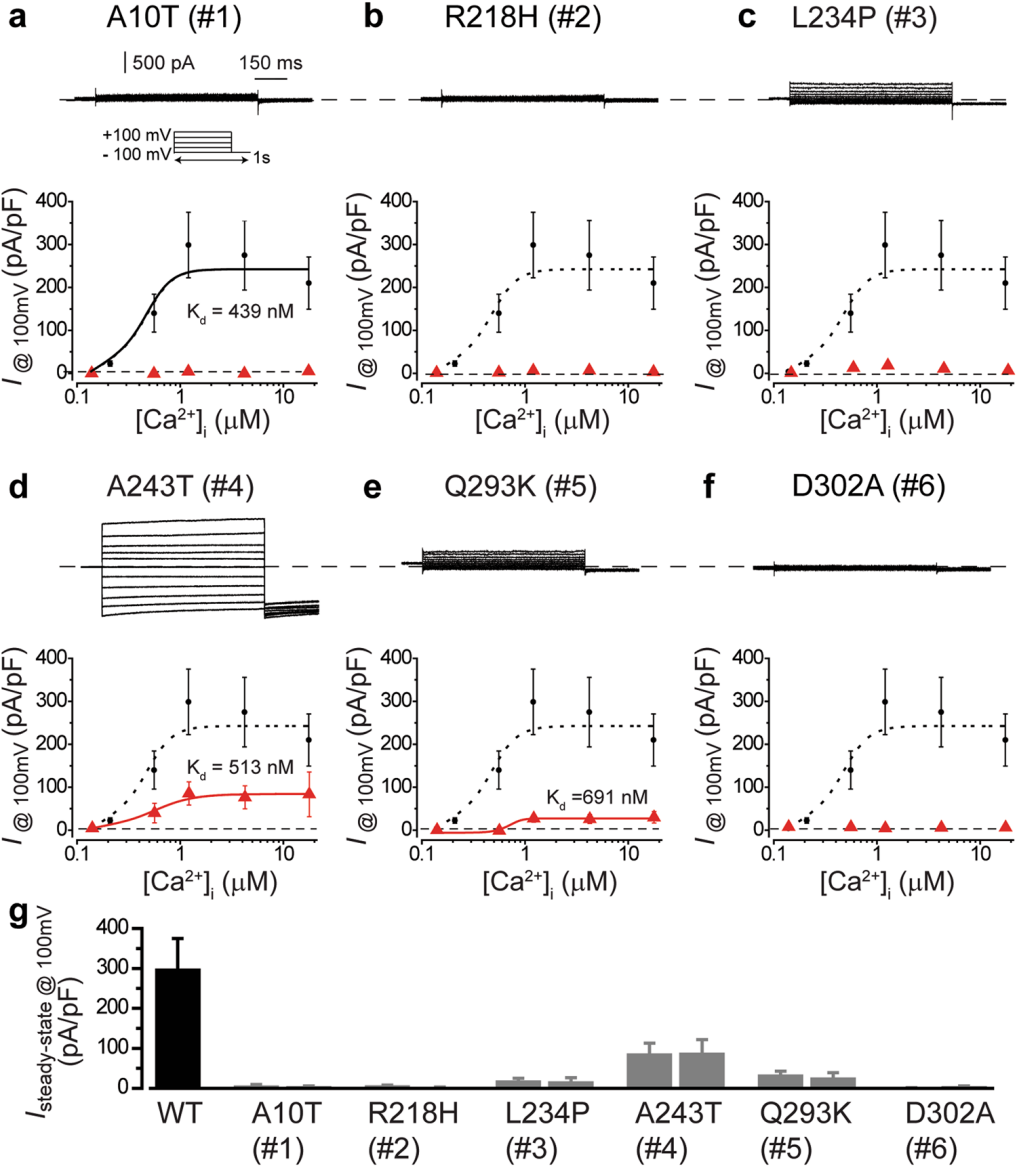
Surface Ca^2+^-dependent Cl^−^ currents in patient-derived iPSC-RPEs. (**a**) Ca^2+^-dependent Cl^−^ currents measured by whole-cell patch clamp in patient-derived iPSC-RPEs bearing the mutation of A10T. *Top*, representative current traces recorded at 1.2 μM [Ca^2+^]_i_. *Inset*, voltage protocol used to elicit currents. *Bottom*, Ca^2+^-dependent current densities, n = 5-6 for each point, compared to WT (•). The WT plot was fitted to the Hill equation. (**b**-**f**) Ca^2+^-dependent Cl^−^ currents measured by whole-cell patch clamp in patient-derived iPSC-RPEs bearing the mutation of R218H (**b**), L234P (**c**), A243T (**d**), Q293K (**e**) and D302A (**f**), respectively. *Top*, representative current traces recorded at 1.2 μM [Ca^2+^]_i_. *Bottom*, Ca^2+^-dependent current densities, n = 5–6 for each point, compared to WT (•). The WT, A234T and Q293K plots were fitted to the Hill equation. (**g**) Comparison of current densities in iPSC-RPEs with WT or mutant BEST1 at 1.2 μM [Ca^2+^]_i_, n = 5–6. Two clonal iPSC-RPEs from each patient. Black, WT. Gray, patient. All error bars in this figure represent s.e.m.

To elucidate the influences of the mutations on the physiological activity of BEST1, we measured Ca^2+^-dependent Cl^−^ current in the patient-derived iPSC-RPEs by whole-cell patch clamp (Fig. 4a–f and Supplementary Fig. S5). Remarkably, tiny currents (<6 pA/pF) were detected in the A10T, R218H and D302A patient-derived iPSC-RPEs at all tested [Ca^2+^]_i_ (Fig. 4a,b,f and Supplementary Fig. S5), suggesting a complete loss of BEST1 channel activity in these mutants. On the other hand, robust currents were detected in the L234P, A243T and Q293K patient-derived iPSC-RPEs, but the current amplitude was significantly reduced compared to that from iPSC-RPE with WT BEST1 (Fig. 4c–e and Supplementary Fig. S5), suggesting a partial loss of function. Moreover, as currents from the A243T and Q293K iPSC-RPEs were large enough for fitting to the Hill equation, their Ca^2+^-sensitivity was calculated: compared to that from the WT iPSC-RPE (K_d_ = 439 nM), Ca^2+^-sensitivity was normal in A243T iPSC-RPE (K_d_ = 513 nM) but significantly right shifted in Q293K iPSC-RPE (K_d_ = 691 nM, Fig. 4d,e), consistent with the structure model in which Q293 but not A243 is involved in Ca^2+^-binding (Fig. 2c). L234P is not expected to affect Ca^2+^-sensitivity, because L234 is localized outside of the Ca^2+^-clasp (Fig. 2c). For each mutation, similar electrophysiological results were obtained from two clonal iPSC-RPEs (Fig. 4g), indicating that the observed defect in Ca^2+^-dependent Cl^−^ current is mutation-specific.

Taken together, our results show that the six mutations analyzed in this work can be classified into two different groups by their phenotypes: complete loss of function (A10T, R218H and D302A), and partial loss of function with normal (A243T and L234P) or decreased (Q293K) Ca^2+^-sensitivity.

### Rescue of *BEST1* dominant mutations by gene supplementation

**Figure 5:**
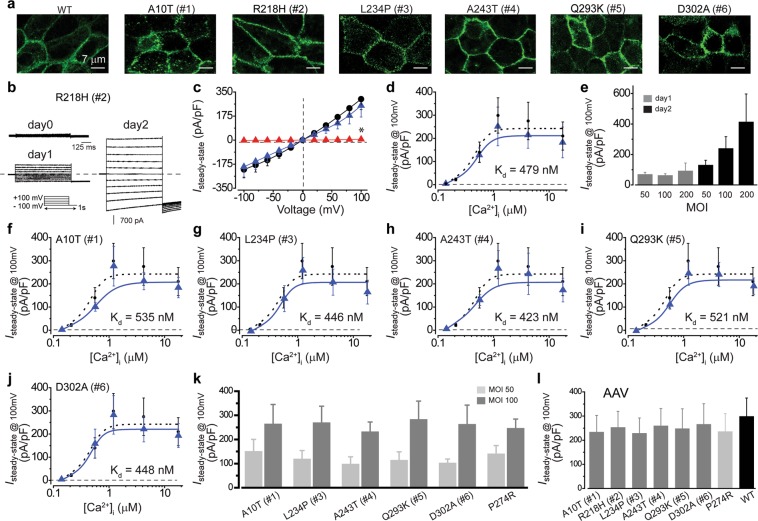
Rescue of patient-derived iPSC-RPEs by WT BEST1 supplementation. (**a**) Confocal images showing the expression of WT BEST1-GFP from BacMam virus in donor-derived iPSC-RPEs. (**b**) Representative current traces recorded from R218H iPSC-RPE (patient #2) supplemented with WT BEST1-GFP at 1.2 μM [Ca^2+^]_i_. (**c**) Current densities in R218H iPSC-RPE supplemented with WT BEST1-GFP (blue triangle) at 1.2 μM [Ca^2+^]_i_, compared to those from un-supplemented R218H (red triangle) and WT (•) iPSC-RPEs. n = 5–6 for each point. *P = 9 × 10^−4^ compared to WT, using two-tailed unpaired Student *t* test. (**d**) Ca^2+^-dependent current densities in R218H iPSC-RPE supplemented with WT BEST1-GFP (blue triangle) compared to those from WT (•) iPSC-RPE. Steady-state current density recorded at + 100 mV plotted vs. free [Ca^2+^]_i_; n = 5–6 for each point. The plots were fitted to the Hill equation. (**e**) Current densities at 1.2 μM [Ca^2+^]_i_ in a second clone of R218H iPSC-RPE supplemented with different dosages of WT BEST1-GFP on BacMam viruses. n = 5–6 for each point. (**f–j**) Ca^2+^-dependent current densities in patient-derived iPSC-RPEs bearing the mutation of A10T (**f**), L234P (**g**), A243T (**h**), Q293K (**i**) and D302A (**j**), supplemented with WT BEST1-GFP (blue triangle), compared to those from WT (•) iPSC-RPE, n = 5–6 for each point. The plots were fitted to the Hill equation. (**k**) Current densities at 1.2 μM [Ca^2+^]_i_ in the second clones of the five BEST1 dominant iPSC-RPEs and a clone of the recessive P274R iPSC-RPE supplemented with different dosages of WT BEST1-GFP from BacMam viruses on day 2. (**i**) Current densities at 1.2 μM [Ca^2+^]_i_ in patient-derived iPSC-RPEs supplemented with WT BEST1 on AAV2 viruses. All error bars in this figure represent s.e.m.

We previously reported that the defective Ca^2+^-dependent Cl^−^ current in patient-derived iPSC-RPEs carrying recessive *BEST1* mutations can be rescued by baculovirus (BV)-mediated supplementation of the WT *BEST1* gene^9^. To investigate if the Ca^2+^-dependent Cl^−^ current is rescuable in iPSC-RPEs bearing *BEST1* dominant mutations, WT BEST1-GFP was expressed from a BV vector in the six patient-derived iPSC-RPEs. Confocal imaging confirmed that WT BEST1-GFP is localized on the plasma membrane of all six patient-derived iPSC-RPEs (Fig. 5a), including A10T, Q293K and D302A iPSC-RPEs in which the membrane localization of endogenous BEST1 is impaired to different degrees (Fig. 3).

For electrophysiological analysis, we first utilized iPSC-RPE carrying the BEST1 R218H mutation to optimize the time course and MOI of virus infection, as R218H is a null mutation with normal membrane localization of endogenous BEST1, representing a “clean” case with strong phenotypes. Ca^2+^-dependent Cl^−^ current measured at 1.2 μM [Ca^2+^]_i_ by whole-cell patch clamp significantly increased from 24 to 48 hours, and in a dose-dependent manner at 48 hours post infection (Fig. 5b–e). A complete rescue of the Cl^−^ current at peak [Ca^2+^]_i_ was observed at 48 hours post infection with a minimum MOI of 100 (Fig. 5c–e, and Supplementary Fig. S5), where Ca^2+^-dependent Cl^−^ currents in a full range of [Ca^2+^]_i_s were also fully restored (Fig. 5d). Consistently, Ca^2+^-dependent Cl^−^ currents in the other five patient-derived iPSC-RPEs were all rescued to a similar level under the same conditions (Fig. 5f–k), regardless of the type or level of deficiency in the endogenous BEST1 function. Immunoblotting results showed that the exogenous BEST1 expression level is comparable to that of the endogenous BEST1 (Supplementary Fig. S4).

Moreover, the rescue efficacy of Ca^2+^-dependent Cl^−^ currents in iPSC-RPEs bearing BEST1 dominant mutations was comparable to that in a previously reported iPSC-RPE with a recessive P274R mutation (Fig. 5k)^9^. Taken together, we concluded that the defect of Ca^2+^-dependent Cl^−^ conductance caused by *BEST1* loss-of-function mutations, either dominant or recessive, is rescuable by BV-mediated supplementation of the WT *BEST1* gene with the same dosage and time course.

To test if *BEST1* supplementation can be mediated by adeno-associated virus (AAV), which has been approved for gene therapy in the human retina^28^, we infected iPSC-RPEs with an AAV serotype 2 (AAV2) viral vector expressing BEST1-T2A-GFP. Consistent with the results from BV-mediated augmentation, Ca^2+^-dependent Cl^−^ currents were restored after AAV infection in iPSC-RPEs bearing either a dominant or recessive *BEST1* mutation (Fig. 5i), providing a proof-of-concept for curing *BEST1*-associated retinal degenerative diseases in both dominant and recessive cases by AAV-mediated gene augmentation.

## Discussion

Here we comprehensively examined six *BEST1* dominant disease-causing mutations (A10T, R218H, L234P, A243T, Q293K and D302A) derived from BVMD patients in an interdisciplinary platform, including whole-cell patch clamp with patient-derived iPSC-RPEs and HEK293 cells expressing the mutant channels, immunodetection of endogenous BEST1 in iPSC-RPEs, structural analyses with human homology models, and virus-mediated *BEST1* gene supplementation. Collectively, our results illustrate the physiological influence of these six dominant mutations on RPE surface Ca^2+^-dependent Cl^−^ current and the BEST1 channel function, provide structural insights into their disease-causing mechanisms, and demonstrate the rescue of BEST1 function in iPSC-RPE via gene supplementation. Notably, the diminished Ca^2+^-dependent Cl^−^ currents in the R218H, L234P and A243T patient-derived iPSC-RPEs are in accord with the deficient Ca^2+^-stimulated Cl^−^ secretion shown by Cl^−^ sensitive fluorescent dyes in these cells^29^.

Previously, we reported that the impaired Ca^2+^-dependent Cl^−^ current in iPSC-RPE derived from an ARB patient bearing a *BEST1* recessive mutation (P274R) was rescuable by BV-mediated supplementation of WT BEST1^9^. Here, we showed that the same strategy, with both BV and AAV2, can be generally applied to restore Ca^2+^-dependent Cl^−^ current impaired by *BEST1* loss-of-function dominant mutations, which can be sub-classified into null (e.g. A10T, R218H and D302A), and partial deficiency with unaffected (e.g. L234P and A243T) or shifted (e.g. Q293K) Ca^2+^-sensitivity. Our results provide a proof-of-concept for the clinical application of *BEST1* gene augmentation, but further optimization is still required: BV has not been approved for human therapy due to its strong adjuvant activity^30^, while the transduction efficiencies of other AAV serotypes (e.g. AAV5 and AAV8) also need to be investigated^31^.

The retina has been the frontier of translational gene therapy in the past 20 years. Recently, the first gene therapy drug, an AAV-based vector carrying a correct copy of the *RPE65* gene, was approved by FDA for treating retinal degenerative Leber congenital amaurosis type 2 (LCA2), which is caused by recessive mutations in *RPE65*^28,32–35^. As another inherited retinal disorder clearly linked to the mutation of a single gene, bestrophinopathy represents an attractive target of gene therapy. However, since the vast majority of known *BEST1* mutations are autosomal dominant, it remains a critical question whether the dominant mutant allele should be purposely suppressed during therapeutic intervention. Our results showed that virus-mediated WT BEST1 gene supplementation restores the diminished Ca^2+^-dependent Cl^−^ currents in patient-derived iPSC-RPEs with the same dose- and time- dependent efficacy regardless of the mutation type (dominant vs. recessive) or deficiency level (null vs. partial), providing one of the first lines of evidence that *BEST1* dominant mutations are rescuable by WT gene augmentation without the need of disrupting/suppressing the mutant allele. In agreement with our findings, a preprint by Sinha *et al*. showed that two more *BEST1* dominant mutations, namely R218C and N296H, can be rescued by lentivirus-mediated gene augmentation in iPSC-RPE cells^36^. Interestingly, a third dominant mutation in that report, A164K, was not responsive to gene augmentation, probably attributed to structural instability as suggested by the authors^36^. Nevertheless, as AAV-mediated subretinal *BEST1* gene augmentation therapy has succeeded in reversing clinically detectable subretinal lesions and diffuse microdetachments in canine *BEST1* recessive mutation models^16^, we predict that the same strategy can be applied to treat patients with either dominant or recessive *BEST1* mutations as long as the mutation causes a loss-of-function.

We recently identified several gain-of-function mutations (e.g. D203A, I205T and Y236C), which significantly enhance BEST1 channel activity^25^. Mechanistically, these mutations dysregulate BEST1 gating at two Ca^2+^-dependent gates, resulting in increased channel opening^25^. Although the pathological basis of elevated BEST1 activity remains unclear, we speculate that knockdown or knockout of the gain-of-function mutant allele is likely necessary in addition to supplementation of the WT *BEST1* gene for restoring normal BEST1 activity in these cases. However, due to the unavailability of patient-derived RPEs bearing gain-of-function mutations, whether the endogenous BEST1 protein level is negatively affected by these mutations remains unclear. Nevertheless, determining whether a patient-derived mutation causes a loss or gain of function is essential for designing the treatment strategy.

Consistent with our previous report that BEST1 is the channel responsible for Ca^2+^-dependent Cl^−^ currents in RPE^9^, the channel activity of heterologously expressed BEST1 mutants in HEK293 cells generally reflects the integrity of Ca^2+^-dependent Cl^−^ currents on the plasma membrane of the corresponding patient-derived iPSC-RPEs (Figs. 2 and 4). Although heterologous expression of WT and mutant channels in HEK293 cells is a standard and powerful approach for functional studies of BEST1, we have noticed two main limitations: firstly, the current density from HEK293 cells transiently transfected with BEST1 is significantly smaller than that from RPE cells; secondly, the Ca^2+^-sensitivity of BEST1-mediated currents in HEK293 cells is left-shifted compared to that in RPEs^9,37^. These discrepancies are likely a result of the intrinsic differences between the two cell types, rather than exogenous vs. endogenous expression of BEST1, as the Ca^2+^-dependent Cl^−^ currents from supplemented WT BEST1-GFP in patient-derived iPSC-RPEs show very similar current density and Ca^2+^-sensitivity to those from endogenous BEST1 in WT iPSC-RPE (Figs. 4 and 5)^9^. We speculate that there are RPE-specific facilitating factor(s) of BEST1.

All three mutations in residues predicted to be involved in Ca^2+^ binding exhibited deficiency in the membrane targeting of endogenous BEST1 in iPSC-RPE, suggesting that the membrane trafficking of BEST1 may be facilitated by Ca^2+^ binding. However, previous studies in transiently transfected HEK293 cells showed that mutations around the Ca^2+^-clasp (N296L, E300Q, D301N, D302N, D303L, D304N, E306Q, and N308D) do not affect channel trafficking^37^. This discrepancy may be attributed to the different cell types and/or mutations in these works.

In summary, we analyzed the clinical, electrophysiological and structural impacts of six *BEST1* dominant mutations, and demonstrated, among the first to our knowledge, the restoration of BEST1 function in iPSC-RPEs bearing dominant mutations by virus-mediated gene augmentation. Importantly, gene augmentation therapy also has great potential to treat other inherited disorders in the retina, such as autosomal dominant retinitis pigmentosa (adRP), which can be caused by mutations in over 25 known genes including *RHO* and *RPE65*^38^. *RHO* is the most frequently mutated gene associated with adRP. AAV-mediated *RHO* augmentation partially rescues retinal degeneration in the well-characterized R23H transgenic mouse model^39,40^, which exhibits loss-of-function evidenced by reduced rhodopsin levels^41–43^. On the other hand, while *RPE65* is mainly associated with LCA, a D477G mutation in it has been linked to adRP^44^. Heterozygous *RPE65 D477G* knock-in mice exhibited reduced isomerase activity and delayed dark adaptation^45^, suggesting a loss-of-function phenotype. Therefore, our results raise the possibility of curing adRP associated with *RPE65* by the FDA approved AAV-RPE65 vector without suppressing the dominant *D477G* mutant allele.

## Methods

### Generation of human iPSC

Using the CytoTune™-iPS 2.0 Sendai Reprogramming Kit (Thermo Fisher Scientific, A16517), donor-provided skin fibroblasts were reprogrammed into pluripotent stem cells (iPSCs). Immunocytofluorescence assays were carried out following the previously published protocol to score iPSC pluripotency^46^. The iPSCs from all the subjects enrolled in this study were characterized by detecting four standard pluripotency markers (SSEA4, Tra-1-60, SOX2 and Nanog). Nuclei were detected by Hoechst staining. All iPSC lines were passaged every 3–6 days while maintained in mTeSR-1 medium (STEMCELL Technologies, 05850). The morphology and nuclear/cytoplasmic ratio were closely monitored to ensure the stability of the iPSC lines. All the iPSC lines were sent for karyotyping by G-banding to verify genome integrity at Cell Line Genetics (Wisconsin, USA).

### Differentiation of iPSC into RPE

iPSC lines were cultured to confluence in 6-well culture dishes pretreated with 1:50 diluted matrigel (CORNING, 356230). For the first 14 days, the differentiation medium consisted of Knock-Out (KO) DMEM (Thermo Fisher Scientific, 10829018), 15% KO serum replacement (Thermo Fisher Scientific, 10829028), 2 mM glutamine (Thermo Fisher Scientific, 35050061), 50 U/ml penicillin-streptomycin (Thermo Fisher Scientific, 10378016), 1% nonessential amino acids (Thermo Fisher Scientific, 11140050), and 10 mM nicotinamide (Sigma-Aldrich, N0636). During day 15–28 of differentiation, the differentiation medium was supplemented with 100 ng/ml human Activin-A (PeproTech, 120–14). From day 29 on, the differentiation medium without Activin-A supplementation was used again until differentiation was completed. After roughly 8–10 weeks, dispersed pigmented flat clusters were formatted and manually picked to matrigel-coated dishes. These cells were kept in RPE culture medium as previously described^47^. It takes another 6–8 weeks in culture for them to form a functional monolayer, which would then be ready for function assays. In addition to well-established classical mature RPE markers (Bestrophin1, CRALBP and RPE65), two more markers (PAX6 and MITF) were also used to validate the RPE fate of the cells. All iPSC-RPE cells in this study were at passage 1. DNA sequencing was used to verify genomic mutations in the mutant iPSC-RPEs.

### Cell lines

Dr. David Yule at University of Rochester kindly gifted HEK293 cells. As HEK293 is included on the International Cell Line Authentication Committee’s list of commonly misidentified cell lines, the cells used in this study were authenticated by short tandem repeat (STR) DNA profiling. The cells were tested negative for mycoplasma contamination, and cultured in DMEM (4.5 g/L glucose, Corning 10013CV) supplemented with 100 μg/ml penicillin-streptomycin and 10% fetal bovine serum.

### Electrophysiology

Using an EPC10 patch clamp amplifier (HEKA Electronics) controlled by Patchmaster (HEKA), whole-cell recordings were conducted 24–72 hours after splitting of RPE cells or transfection of HEK293 cells. 1.5 mm thin-walled glass with filament (WPI Instruments) were pulled and fashioned to micropipettes, and filled with internal solution containing (in mM): 130 CsCl, 10 EGTA, 1 MgCl_2_, 2 MgATP (added fresh), 10 HEPES (pH 7.4, adjusted by CsOH), and CaCl_2_ to obtain the desired free Ca^2+^ concentration (maxchelator.stanford.edu/CaMgATPEGTA-TS.htm). Series resistance was usually 1.5–2.5 MΩ. There was no electronic series resistance compensation. External solution contained (in mM): 140 NaCl, 15 glucose, 5 KCl, 2 CaCl_2_, 1 MgCl_2_ and 10 HEPES (pH 7.4, adjusted by NaOH). Solution osmolarity was between 310 and 315. A family of step potentials (−100 to + 100 mV from a holding potential of 0 mV) were used to generate I-V curves. Currents were sampled at 25 kHz and filtered at 5 or 10 kHz. Traces were acquired at a repetition interval of 4 s^48^. All experiments in this study were carried out at ambient temperature (23 ± 2 °C).

### Immunoblot Analysis

Cell pellets were extracted by the M-PER mammalian protein extraction reagent (Thermo Fisher Scientific, 78501) or Mem-PER Plus membrane protein extraction kit (Thermo Fisher Scientific, 89842) with proteinase inhibitors (Roche, 04693159001), and the protein concentration was quantified by a Bio-Rad protein reader. After denaturing at 95 °C for 5 min, the samples (20 μg) were run on 4–15% gradient SDS-PAGE gel at room temperature, and wet transferred onto nitrocellulose membrane at 4 °C. The membranes were incubated in blocking buffer containing 5% (w/v) non-fat milk for 1 hour at room temperature, and subsequently incubated overnight at 4 °C in blocking buffer supplemented with primary antibody. Primary antibodies against the following proteins were used for immunoblotting: CRALBP (1:500 Abcam, ab15051), RPE65 (1:1,000 Novus Biologicals, NB100-355), β-actin (1:2,000 Abcam, ab8227), BESTROPHIN-1 (1:500 Novus Biologicals, NB300-164), His (1:1,000 Fisher Scientific, PA1983B) and Myc (1:1,000 Fisher Scientific, PA1981). Fluorophore-conjugated mouse and rabbit secondary antibodies (LI-COR Biosciences, 925–68070 and 925–32213, respectively) were used at a concentration of 1:10,000 and an incubation time of 1 h at room temperature, followed by infrared imaging.

### Immunoprecipitation

HEK293 cells cultured on 6-cm dishes were co-transfected with pBacman-BEST1(WT)-CFP-myc and pBacman-BEST1(mutant or WT)-YFP-His at 1:1 ratio using PolyJet™ *In Vitro* DNA Transfection Reagent (SignaGen Laboratories, SL100688) following the manufacturer’s standard protocol. 48 h later, cells were harvested by centrifugation at 1000 x g for 5 min at room temperature. Cell pellets were lysed in pre-cooled lysis buffer (150 mM NaCl, 50 mM Tris, 0.5% IGEPAL® CA-630, pH 7.4) supplemented with protease inhibitor cocktails (Roche, 04693159001) for 30 min on ice, and then centrifuged at 13,000 rpm for 12 min at 4 °C. The supernatants (300 μg) was collected and mixed with 2 μg Myc monoclonal antibody (Thermo Fisher Scientific, MA1–980). After rotating overnight at 4 °C, the mixture was incubated with Dynabeads M-280 sheep anti-mouse IgG (Thermo Fisher Scientific, 11202D) for 5 h at 4 °C. After thorough washing of the beads, bound fractions were eluted in 1x SDS sample buffer (Biorad, 1610747) by heating for 10 min at 75 °C. Proteins were then resolved by SDS-PAGE and analyzed by immunoblotting.

### Immunofluorescence

RPE cells cultured on coverslips were washed with PBS twice, and fixed in 4% sucrose and 4% paraformaldehyde at room temperature for 45 min. The fixed cells were permeabilized in PBS containing 0.25% Triton X-100 at room temperature for 10 min. In order to block non-specific binding sites, the samples were incubated with PBS containing 5% BSA at room temperature for 1 h. Primary antibodies were diluted in blocking solution as follows: mouse anti-bestrophin 1 (Novus Biologicals, NB300-164), 1:1000; rabbit anti-collagen IV (Abcam, ab6586), 1:500. The samples were incubated with primary antibody in blocking solution overnight at 4 °C. The next day, the samples were washed with PBS thrice. Then, Alexa Fluor 488 conjugated donkey anti-mouse IgG (Thermo Fisher Scientific, A-21202) and Alexa Fluor 647 conjugated donkey anti-rabbit IgG (Thermo Fisher Scientific, A-31573) were diluted in blocking solution and incubated with cells at room temperature for 1 h. Unbound secondary antibody was washed away with PBS thrice. The samples were then incubated with Hoechst 33342 diluted to 1 μg/ml in PBS at room temperature for 10 min. After thorough washing, coverslips were mounted onto ProLong Diamond Antifade Mountant (Thermo Fisher Scientific, P36966). An Olympus laser scanning confocal microscope was used to acquire images, which were then processed in Fiji (https://fiji.sc/). Background was subtracted by the rolling ball method in Fiji with a radius of 50 pixels.

### Virus

BacMam baculovirus bearing BEST1-GFP was made in-house as previously described^49^, and added to RPE culture medium at desired MOI (50–200). High titer AAV2 virus (1 × 10^12^ GC/ml) bearing a CMV promoter driven BEST1-T2A-GFP expression cassette was purchased from Applied Biological Materials.

### Molecular cloning

Point mutations in BEST1 were made by site-directed mutagenesis PCR with the In-fusion Cloning Kit (Clontech). All constructs were fully sequenced.

### Transfection

20–24 h before transfection, HEK293 cells were lifted by incubation with 0.25% trypsin at room temperature for 5 min, and split into fresh 3.5-cm culture dishes at proximately 50% confluency. PolyJet transfection reagent (SignaGen SL100688) was utilized to transfect HEK293 cells with plasmids bearing the WT BEST1 or desired mutant (1 μg). 6–8 h later, the transfection mix was removed, and cells were rinsed with PBS once and cultured in supplemented DMEM. 24 h post transfection, cells were lifted again by trypsin treatment and split onto fibronectin-coated glass coverslips for patch clamp^50^.

### Electrophysiological data and statistical analyses

With Patchmaster (HEKA), Microsoft Excel and Origin, patch clamp data were analyzed off-line. Statistical analyses were conducted using built-in functions in Origin. For comparisons between two groups, statistically significant differences between means (*P* < 0.05) were determined using Student’s *t* test. Data are presented as means ± s.e.m^51^.

### Homology modeling of human BEST1

A homology model for BEST1 was generated using the Swiss-Model server from the chicken Best1 crustal structure^21^. All figures were made in PyMOL.

### Patients and clinical analysis

The healthy control donor (WT *BEST1*) and patients (mutant *BEST1*) all underwent a complete ophthalmic examination by a retinal physician in the Department of Ophthalmology, Columbia University Medical Center/New York Presbyterian Hospital. This included funduscopy, best-corrected visual acuity, and slit-lamp biomicroscopy. Patients underwent OCT and color fundus photography^52,53^. Skin biopsy samples were obtained from the healthy control donor and patients, and processed and cultured as previously described^46^. For these procedures, all of which were approved by Columbia University Institutional Review Board (IRB) protocol AAAF1849, patients 2, 4–6 and the parent(s)/legal guardian(s) of patients 1 and 3 provided written informed consent. All methods were performed in accordance with the relevant regulations and guidelines.

## Supplementary information

Supplementary information

## Data Availability

Data supporting the findings of this manuscript are available from the corresponding author upon reasonable request.
