# Supplementary material for: Investigation and Restoration of BEST1 Activity in Patient-derived RPEs with Dominant Mutations
Source: Sci Rep. 2019 Dec 13;9:19026. doi: 10.1038/s41598-019-54892-7 (PMC6910965; doi:10.1038/s41598-019-54892-7)
Supplement: Supplementary file 1 — Supplementary information [file 41598_2019_54892_MOESM1_ESM.pdf]

Supplementary Information

Investigation and Restoration of BEST1 Activity in Patient-derived RPEs with Dominant Mutations

Changyi Ji, Yao Li, Alec Kittredge, Austin Hopiavuori, Nancy Ward, Peng Yao, Yohta Fukuda, Yu Zhang, Stephen H. Tsang and Tingting Yang

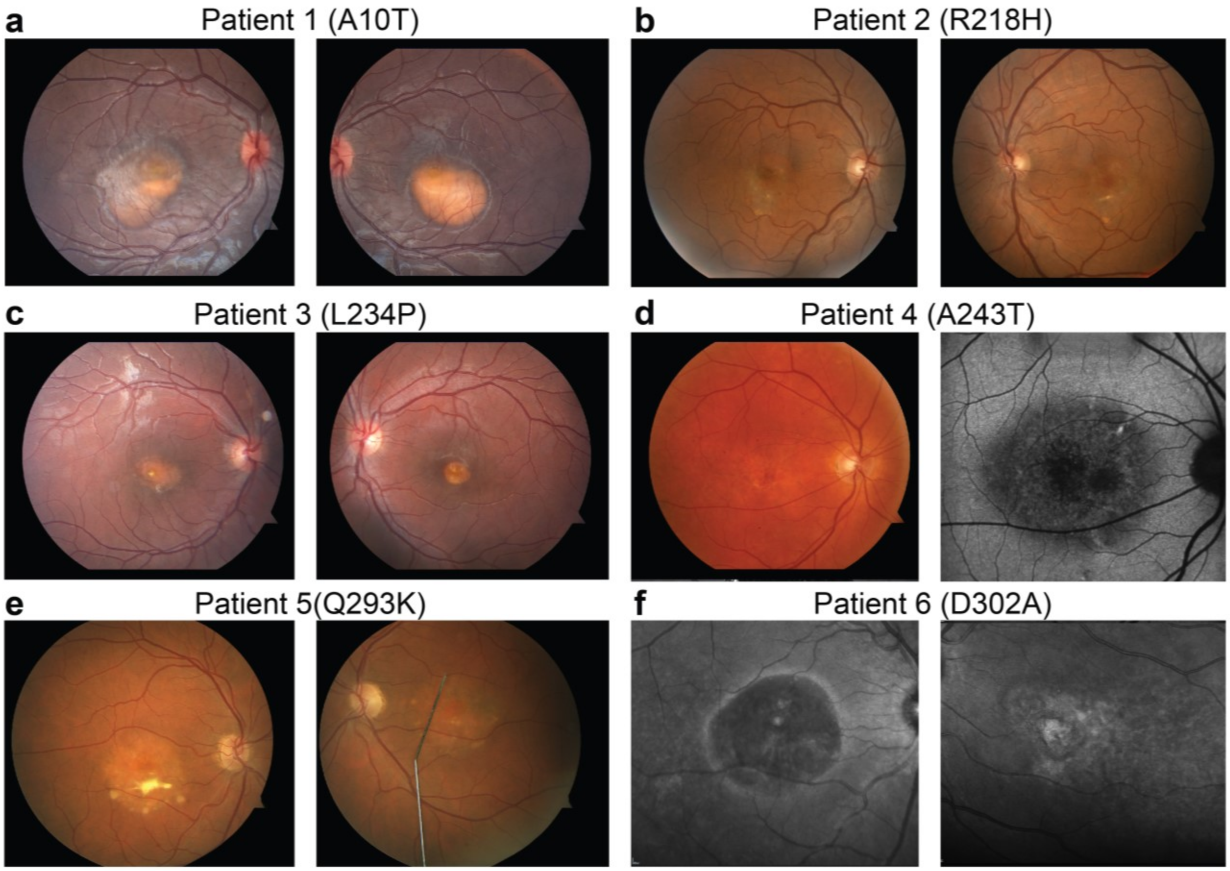

Figure S1. Fundus photographs from patients. (**a**-**f**) Fundus photographs from indicated patients, right and left eye, respectively, except for **d**, in which both photographs are from the right eye of the patient.

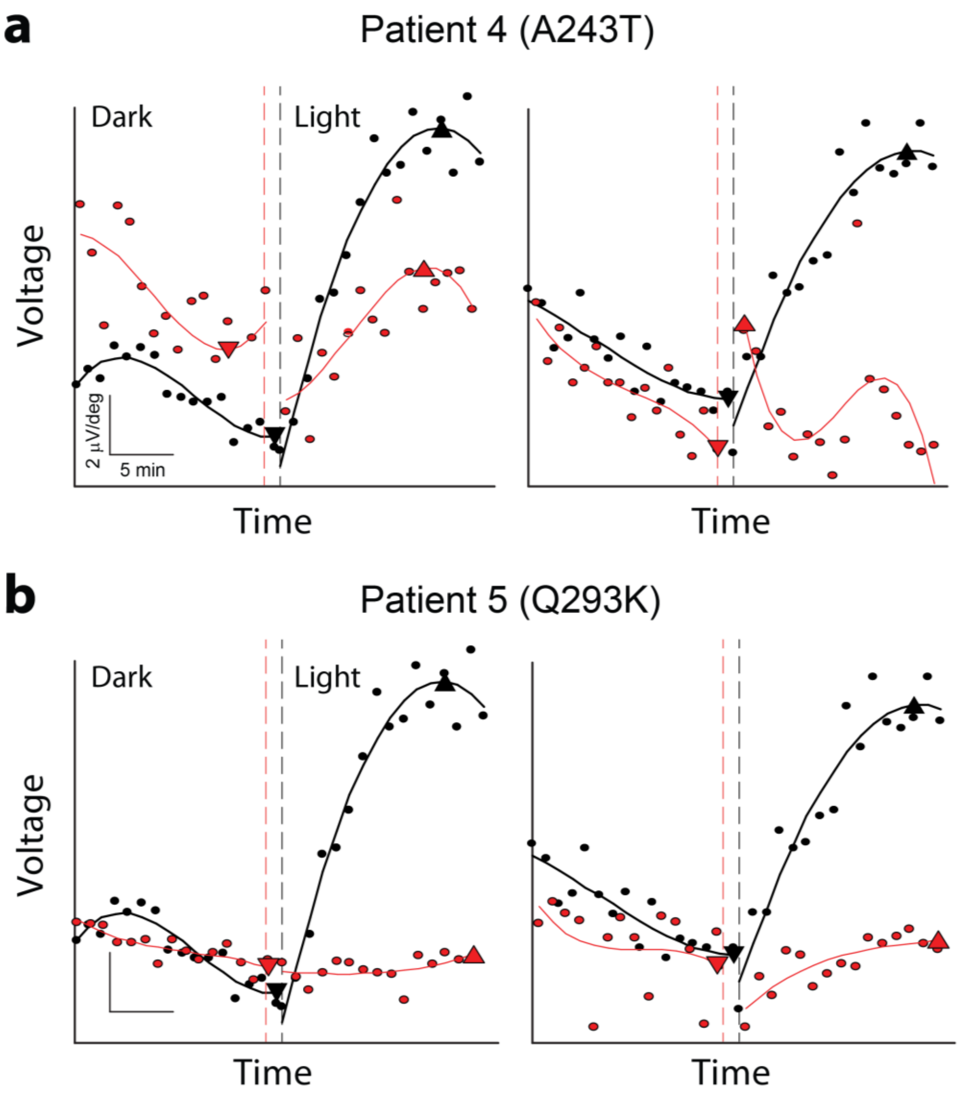

Figure S2. The lack of light rise in EOG profiles of patients #4 and #5. (**a-b**) The EOG profiles of *BEST1* p.A243T (**a**) and p.Q293K (**b**) patients (red) were compared to that of a *BEST1* WT (black) person. Right and left eye, respectively.

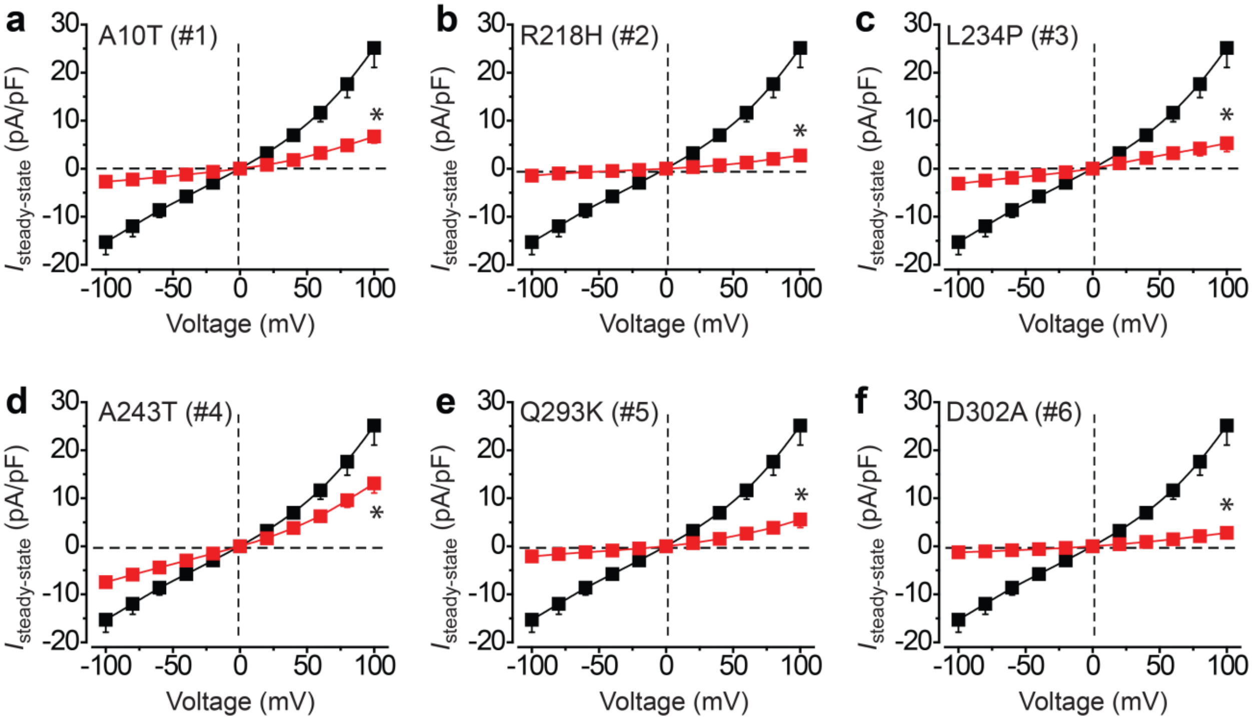

Figure S3. Channel activity of BEST1 mutants in HEK293 cells. (**a-f**) Ca^{2+}-dependent Cl^- current densities at 1.2 μM $[Ca^{2+}]_i$ in HEK293 cells transiently expressing indicated BEST1 mutants (red), compared to the WT (black), n= 5-6 for each point. **P* < 0.05 compared to WT cells, using two-tailed unpaired Student *t* test. Controls are from the same set of data. All error bars in this figure represent s.e.m.

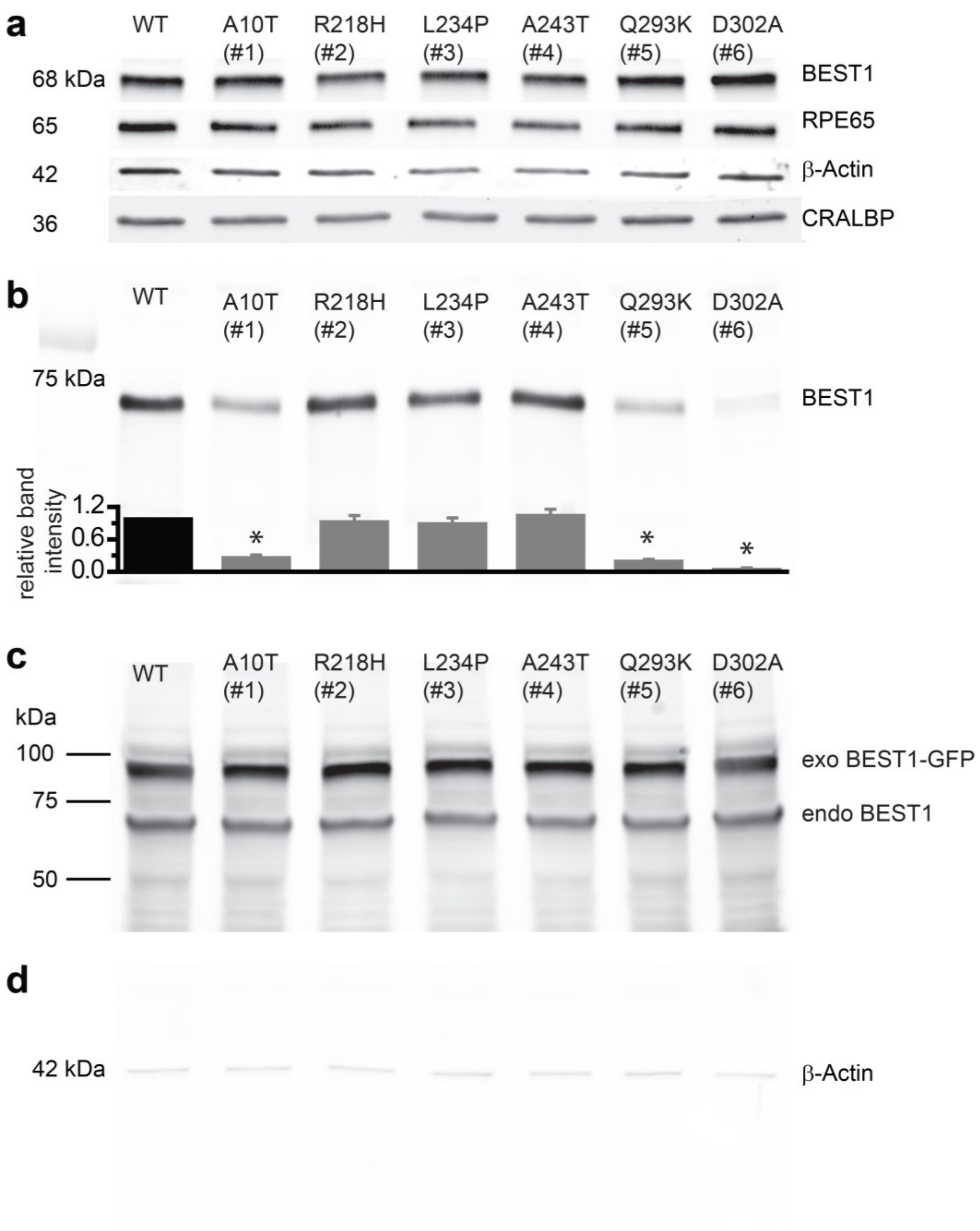

Figure S4. Protein expression in RPE cells. (**a**) Marker proteins in WT and patient-derived iPSC-RPEs. The expression of RPE-specific proteins BEST1, RPE65 and CRALBP were detected by immunoblotting. Two gels/blots were prepared from the same cell lysate of each iPSC-RPE to detect BEST1 + CRALBP, and RPE65 + β-Actin, respectively. Full-length blots are shown in Fig. S6. (**b**) *Top*, cell surface expression of BEST1 in iPSC-RPEs was detected by immunoblotting. Membrane extractions were generated from the same batch of cell pellets as in **a**. *Bottom*, quantitation of the levels of BEST1 in plasma membrane from 3 independent experiments. Data were normalized to the loading control global BEST1 and then compared to WT. **P* < 0.05 compared to WT cells, using two-tailed unpaired Student *t* test. All error bars in this figure represent s.e.m. (**c**) Baculovirus supplemented exogenous BEST1-GFP (WT) and endogenous BEST1 (WT/mutant) in whole cell lysate were detected by immunoblotting. (**d**) The loading control β-Actin from the same set of samples in **c** was detected by immunoblotting.

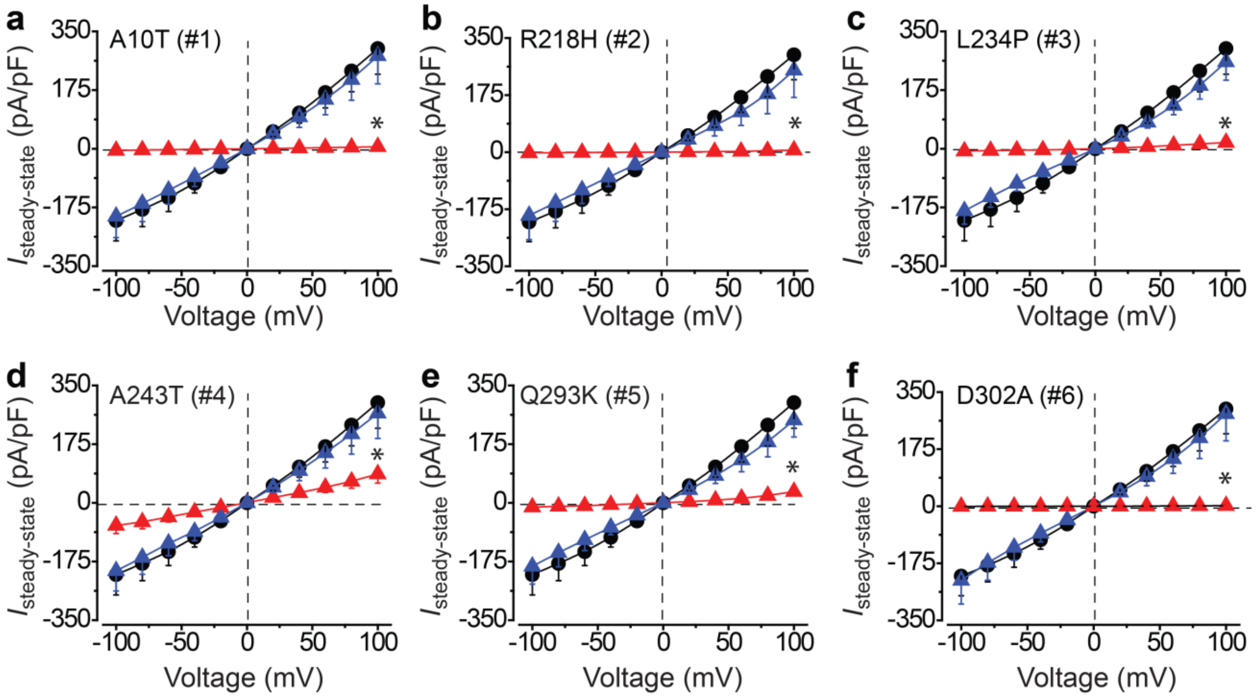

Figure S5. Ca^{2+}-dependent Cl^- currents in patient-derived iPSC-RPEs. (**a-f**) Ca^{2+}-dependent Cl^- current densities at 1.2 µM $[Ca^{2+}]_i$ in indicated patient-derived iPSC-RPEs supplemented with WT BEST1-GFP (▲), compared to un-supplemented mutant (▲), and WT (●) iPSC-RPEs. n= 5-6 for each point. **P* < 0.05 compared to WT cells, using two-tailed unpaired Student *t* test. Controls are from the same set of data. All error bars in this figure represent s.e.m.

Fig. 2b

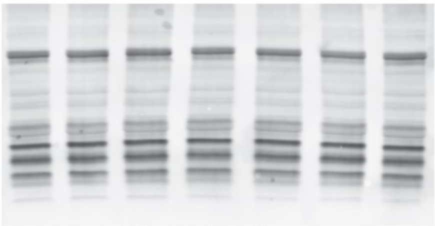

His IB input

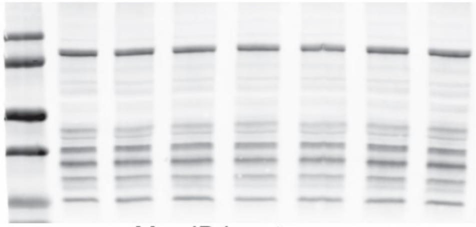

Myc IB input

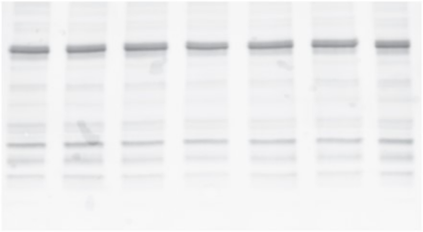

Myc IP His IB

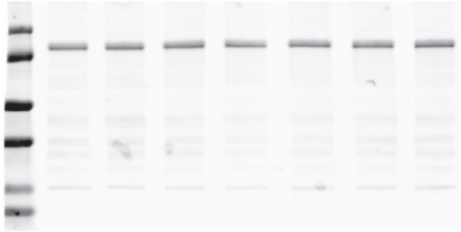

Myc IP Myc IB

Fig. S4

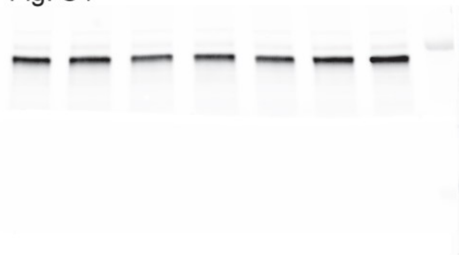

BEST1

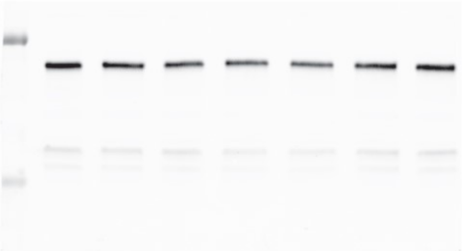

RPE65

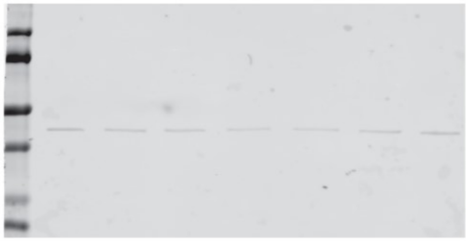

β-Actin

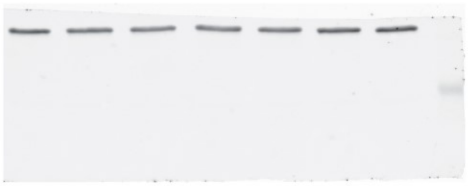

CRALBP

Figure S6. The full-length blots in Figure 2b and Supplementary Fig. S4a.
